# Neighbourhood Society: Nesting Dynamics, Usurpations and Social Behaviour in Solitary Bees

**DOI:** 10.1371/journal.pone.0073806

**Published:** 2013-09-16

**Authors:** Kateřina Černá, Monika Zemenová, Lenka Macháčková, Zdislava Kolínová, Jakub Straka

**Affiliations:** 1 Department of Zoology, Faculty of Science, Charles University in Prague, Prague, Czech Republic; 2 Department of Cybernetics, Faculty of Electrical Engineering, Czech Technical University in Prague, Prague, Czech Republic; University of Freiburg, Germany

## Abstract

Intraspecific cleptoparasitism represents a facultative strategy advantageous for reducing time and energy costs. However, only a few studies about nesting dynamics have described intraspecific cleptoparasitic behaviour in obligate solitary bees. We focused on nesting dynamics with the characterisation of nest owner replacements and frequency of true usurpation in four aggregating species belonging to different phylogenetic lineages – *Andrena vaga* (Andrenidae), *Anthophora plumipes* (Apidae), *Colletes cunicularius* (Colletidae), and *Osmia rufa* (Megachilidae). Our study, based on the regular observation of individually marked females, shows that nest owner replacement affects 10–45% of nests across all of the studied species and years. However, 39–90% of these nests had been abandoned before owner change and thus true nest usurpations represent only a part of observed nest replacement cases. Females tend to abandon their nests regularly and found new ones when they live long enough, which is in accordance with risk-spreading strategy. We suggest that the original facultative strategy of observed solitary bees during nest founding is not cleptoparasitism per se but rather reuse of any pre-existing nest (similar to “entering” strategy in apoid wasps). This is supported by gradual increase of nests founded by “entering” during the season with an increase in the number of available nests. Although the frequent reuse of conspecific nests results in frequent contact between solitary females, and rarely, in the short-term coexistence of two females in one nest, we detected unexpectedly low level of conflict in these neighbourhood societies. We suggest that nesting dynamics with regular nest switching and reusing reduces long-term and costly intraspecific aggression, a key factor for the origin and evolution of sociality.

## Introduction

Social behaviour is a widely studied phenomenon, fascinating researchers for its cooperative principle, presence of communication, rise of intraspecific tolerance and hidden conflicts [1]. In comparison to social species, the social aspects of obligate solitary species are not commonly studied in detail. Solitary species nesting in aggregations represent one of the exceptions. Hymenoptera often nest in aggregations because places with optimal nesting conditions are limited and/or living in aggregations provide protection from parasites and predators. Here, we do not deal with all the possible factors influencing the rise of aggregations in solitary bees as there are comprehensive reviews on this topic (e.g., [2]–[6]), but we look at the aggregations as a potential prior state for the evolution of sociality [7]. Although the significance of nesting aggregations in the evolution of bee sociality has been acknowledged in the literature [7], behaviour of their members is usually considered rather simple and social aspects of such behaviour are often neglected: “If a number of organisms are close together, yet do not influence one another, one may speak of the group as an aggregation but not as a society” [8].

Females nesting in aggregations, although solitary, often come into contact with other conspecific females [9]. Most of these interactions are associated with the nest because constructing and provisioning a nest always requires an investment of time and energy. Such investment is prone to be exploited by cleptoparasitic females, that can either steal the provision from outside or inside of the nest, usurp the nest structure after removing the host cells, discard the provision and use the empty nest cells, usurp the nest including provision or parasitize the brood ( = cuckoo behaviour) [10]. When a female uses an already constructed nest structure, supplies or brood cells, she can spare much energy and time and can increase her fitness significantly [10]. However, such a cleptoparasitic female must enter the active nest of conspecific female and she thus risks a conflict with the nest owner, which can be in case of aculeate Hymenoptera very dangerous because of an efficient weapon – the sting [9]–[11].

Intraspecific cleptoparasitism is closely associated with nest-founding dynamics. Every time a female founds a nest, she can decide either to found and provision a new nest or to parasitize the nest of a conspecific female [12]. Intraspecific cleptoparasitism (sensu Field [10]) thus typically represents a facultative strategy that is advantageous under certain conditions [13]. Ward and Kukuk [14] showed that nest usurpation in the solitary phase of some species of primitively eusocial genus *Lasioglossum*, when the social species does not behaviourally differ from its solitary relatives, can represent an evolutionarily stable strategy, with different factors triggering the cleptoparasitic behaviour, such as the probability of nest disturbance or death during provisioning, the soil quality or the owner-usurper motivation asymmetry. The probability of adopting a cleptoparasitic strategy might further depend on other factors, such as the availability of suitable nesting places [13], food availability [15], time of the day and temperature [12] or phenotype [16], [17].

Brockmann and Dawkins and Brockmann et al. [18], [19] studied nest founding strategies in the solitary apoid wasp *Sphex ichneumoneus* in detail. Interestingly, these authors showed that *Sphex ichneumoneus* individually chooses between strategies of “digging” (constructing a completely new nest) and “entering” (inhabiting any existing nest) during each nest founding. Their model implies that usurpations and the incidental cohabiting of one nest by two females (commonly interpreted as nestmate joining) might be only by-products of not being able to distinguish the active nests from abandoned ones when using the “entering” strategy, at least in this species. It represents an interesting addition to the condition-based cleptoparasitic decision making during nest founding described above.

As mentioned above, Field [10] distinguished six types of intraspecific cleptoparasitism that are applicable to all of the aculeate Hymenoptera. Intraspecific cleptoparasitism is common in eusocial bee species during the solitary phase in form of usurpations [15], [20], [21] or as a form of intraspecific social (brood) parasitism [22], [23]. In solitary bee species, nest usurpation with or without the discarding and brood parasitism (cuckoo behaviour) had been documented only in several species from four bee families - the Megachilidae [13], [24]–[27], the Apidae [28]–[30], the Colletidae [31] and the Halictidae [17] (see also [10], [32]).

Although Michener [8] noted that nest usurpations are “not uncommon” for solitary and other bees, this is not supported by published data in our opinion. Therefore, we decided to focus on the nesting dynamics and characterisation of nest usurpations in most of the bee families from the main phylogenetic lineages (Apidae, Andrenidae, Colletidae and Megachilidae) in detail. Because we recognised that true usurpations represent only one of the possible situations that occur during owner change within the nest, we decided to use the more general term nest owner replacement instead of traditionally used term usurpation for any detected ownership change in the nests. The nest owner replacements were detected by individual marking and regular daily monitoring of marked individuals and their nests. The frequent contact given by high frequency of nest owner replacements and low aggressiveness detected among individuals in these neighbourhood societies show the possible evolutionary significance of intraspecific cleptoparasitism in solitary bees for the evolution of sociality, because described situation within the principle lineages of obligate solitary bees might represent an analogy to the behaviour of solitary species at the beginning of social evolution.

## Methods

This study is based on the observations of four univoltine spring species from four bee families (Hymenoptera: Apoidea): *Andrena vaga* (Andrenidae), *Anthophora plumipes* (Apidae), *Colletes cunicularius* (Colletidae) and *Osmia rufa* (Megachilidae). Studied species considerably differ in nest construction. While *A. vaga* and *C. cunicularius* built deep nests in sandy soil, *A. plumipes* dug shallow nests in dry soil protected from the rain (in our case under the staircase) and *O. rufa* nested in pre-existing cavities in the wall, where it brought mud as construction material. All of the species were observed for 1–3 entire nesting seasons in 2007–2010 at different localities in the Czech Republic (Table 1). All the sites are located outside national parks or any other protected areas, no specific permissions were thus required for the behavioural observations. The field studies also did not involve endangered or protected species according to Czech or international law.

**Table 1:** Summary of the observation period, locality and number of observed females used for behavioural observations in different seasons.

Species	Date	Locality	GPS	N
*Andrena vaga*	23.3.–25.4.2007	Čelákovice	50°11′0.5″N, 14°46′13.9″E	381
*Andrena vaga*	28.3.–10.5.2008	Čelákovice	50°11′0.5″N, 14°46′13.9″E	232
*Anthophora plumipes*	3.4.–2.6.2008	Praha - Strahov	50°4′47.7″N, 14°23′32.7″E	83
*Anthophora plumipes*	20.4.–28.5.2009	Praha - Strahov	50°4′47.7″N, 14°23′32.7″E	97
*Anthophora plumipes*	7.4.–9.6.2010	Praha - Strahov	50°4′47.7″N, 14°23′32.7″E	143
*Osmia rufa*	8.6.–24.6.2010	Praha - Černý Most	50°6′08.3″N, 14°34′14.4″E	26
*Colletes cunicularius*	30.3.–27.4.2010	Čelákovice	50°10′26.1″N, 14°45′23.1″E	103

N – number of individually marked observed females.

All of the females and nests on the nesting site were individually marked. We marked females with oil-based markers, using three different colour spots (two thoracic and one abdominal or vice versa) for each female. The nests were marked using pins with a colour combination corresponding to the female owner. We monitored the changes in nest ownership and described the females’ activities on the nesting site every day during the season (except for rainy days, when the activity of bees is minimal). When we observed a different female activate in the nest (for different activity patterns see [33]), we considered it as the nest owner replacement, and the nest was re-marked by a colour combination corresponding to a new owner.

The raw data that were obtained during the field observations were stored in a relational database, PostgreSQL Database Server 8.3, and we used the SQL language to generate the desired information. During the data analysis, we distinguished the existence of four different states that were hidden in the nest owner replacement situations detected during field observations: 1) True usurpation (TU) occurred when the host female actively provisioning the nest was replaced by another female that began using this nest. On specific occasions the original nest owner did not left the nest immediately after the true usurpation resulting in cohabitation of both the usurper and the previous owner in the same nest for limited time (situations TU-PE). Usurper stayed in the nest for at least two days and was observed to provision the nest. 2) Occupation of the abandoned nest (OA) occurred when the last activity of the original female was documented two or more days before a new female occupied the nest. 3) Failed usurpation (FU) occurred when the original owner either continued to live in the original nest or left the nest, but the usurper was not observed occupying the nest the following day(s) after the usurpation attempt. 4) Failed occupation of an abandoned nest (FOA) occurred when a female attempted to occupy an empty nest but was not active inside the nest for more than a few hours and was not observed in the nest in following day(s). However, bees that failed to usurp or occupy the empty nest stayed in the nest longer than during occasional orientation mistakes, which usually last just a few minutes. A one day lack of activity in the nest does not have to mean nest abandonment because the females are not active every day regardless of the weather conditions [33]. For this reason, we used two days as a criterion to state that there was no activity of an individual in the nest.

We further described the fate of the original owners after situations 1–4 to determine how different individuals and species react to threatening situations. The original owner could a) disappear (DI - we did not detect any activity by the original owner at the nesting site afterward), b) move to a new nest (MO), c) be nest-less (NL - a female was observed for several days at the nesting site but did not found a new nest) and d) persist in the original nest (PE). The former owner of an abandoned nest could further be e) nest-less for more than two days after nest abandonment and then found a new nest (NLMO); such delayed behaviour has not been observed in other situations (a–d).

Because we evaluated the nest-founding strategy in studied species as “digging”/“entering” sensu Brockmann [19], we were further interested in the distribution of the “entering” strategy during the season. We analysed the dependence of the “entering” strategy frequency on the order of days in the season (day 1 = date of first emergence of bees at the beginning of the nesting season) in each species and year using the quasibinominal glm model in the program R 2.14.0 [34]. We further tested correlation between the order of the days and the number of available empty nests (caused by nest abandonment, or death of the owner) using R 2.14.0, because the number of available nests could be an important factor for adopting the “entering” strategy. The correlation between the time and number of empty nests was not tested in *Osmia*, *Colletes* and *Andrena* 2007, because we studied only a part of a large nesting area and we thus did not know the exact numbers of available empty nests.

We found that females often built more than one nest during their life and founding a new nest always resulted in abandoning their old nests (nest was claimed as abandoned the same day, when the original owner founded a new nest elsewhere). The reason for the moving was generally unknown (not caused either by usurpation or by any observed disturbance). To test the hypothesis that females abandon their nests regularly (move) after a specific time, we examined the time intervals that each female spent in each nest and the relationship between the number of nests founded by one female and the longevity of the female: 1) We compared the time spent in subsequent nests by all of the females using a one-way ANOVA, and 2) we used a two-tailed standard two-sample t-test to test the hypothesis that females with more than one nest live significantly longer than females with only one nest. We used the program R 2.14.0 [34] and a significance threshold of *α* = 0.05 for both analyses. We omitted the data from *Osmia* in these two analyses because the sample size was not sufficient.

Finally, we quantified all of the cases of observed intraspecific aggressiveness that occurred at the nesting site, usually in close proximity of the nest entrances (such as biting or stinging with audible buzzing) during the observation period for all of the species (across all of the seasons for each species) and compared it with the number of true and failed usurpations, in which we assumed the certain presence of intraspecific contacts between females, and which thus represents the estimate of the minimal expected number of aggressive incidents. Statistical analyses were not performed because of the extremely low number of observed aggressive attacks.

## Results and Discussion

**Figure 1:**
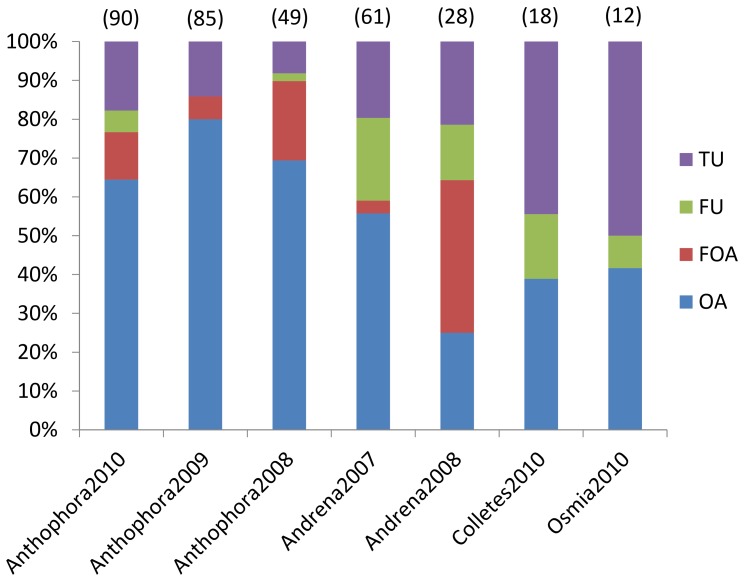
Proportions of different situations during nest owner replacement. The figure shows proportion in different seasons and species. TU – true usurpation, FU – failed usurpation, FOA – failed occupation of abandoned nest, OA – occupation of abandoned nest.

Our study shows that nest owner replacements are very common in all of the studied species in all of the observed years. About 10–45% of the nests changed owners during the season (Table 2). However, when the data were analysed in detail, we recognised that most of the nests that changed owners in *Anthophora* and *Andrena* and approximately 40% of the nests that changed owners in *Colletes* and *Osmia* had been abandoned by the female owners (OA+FOA) before owner change. Only 2–23% of all of the nests were truly usurped (TU) (Fig. 1, Table 2) and true usurpations thus represent rather minor part of observed nest owner replacement situations. In *Andrena*, *Anthophora* and *Osmia*, the nest owner replacement usually occurred quickly during true usurpations, although we observed a few occurrences when both females (usurper and owner) provisioned the nest simultaneously for several days (situation TU-PE in Table 2), which can be interpreted as temporary joining. However, we purposely decided to call such situation TU-PE rather than joining, because we consider this behaviour to be unintentional and accidental contrary to the traditional definition of joining, i.e. a facultative strategy increasing the individuals fitness [7]. Interestingly, the true usurpation resulted in the situation TU-PE in 6 out of 9 cases in *Collete*s. When we analysed the further fate of TU-PE nests and bees in all of the studied species (N = 16), we found that although both the original owner and usurper provisioned the nest after usurpation for a few days, the first (N = 5), the latter (N = 6) or both (N = 5) eventually left the nest. We did not recognise any difference between studied species in this event; neither the original owner nor the usurper seems to be benefited. The refugees usually founded a new nest or disappeared in a few days.

**Table 2:** Quantification and proportions of different situations during usurpation of the nests in different seasons and species (OA = occupation of abandoned nest, FOA – failed occupation of abandoned nest, TU – true usurpation, FU – failed usurpation, DI – disappear, MO – move, NL – nest-less, NLMO – nest-less for a few days then move, PE – persist in the original nest).

Type ofusurpation	Owner afterusurpation	*Anth*2010	*Anth*2009	*Anth*2008	*Andr*2007	*Andr*2008	*Colletes*2010	*Osmia*2010
OA	DI	22	11	15	16	1	2	5
OA	MO	15	34	10	12	0	5	0
OA	NL	20	8	9	1	3	0	0
OA	NLMO	1	15	0	5	3	0	0
	**sumOA**	**58**	**68**	**34**	**34**	**7**	**7**	**5**
FOA	DI	1	1	7	0	1	0	0
FOA	MO	2	4	2	2	1	0	0
FOA	NL	7	0	1	0	7	0	0
FOA	NLMO	1	0	0	0	2	0	0
	**sumFOA**	**11**	**5**	**10**	**2**	**11**	**0**	**0**
FU	NL	2	0	0	0	0	2	0
FU	DI	1	0	0	2	0	0	0
FU	MO	0	0	0	3	0	0	0
FU	PE	2	0	1	8	4	0	0
	**sumFU**	**5**	**0**	**1**	**13**	**4**	**2**	**0**
TU	DI	7	1	1	2	0	1	4
TU	MO	3	8	3	5	1	2	1
TU	NL	5	2	0	2	2	0	0
TU	PE	1	1	0	3	3	6	2
	**sumTU**	**16**	**12**	**4**	**12**	**6**	**9**	**7**
Total nest number		198	234	150	500	286	126	31
Nests with owner change (OA+FOA+TU+FU)		90	85	49	61	28	18	12
= % (of total nest number)		45%	36%	33%	12%	10%	14%	39%
Abandoned nests (moving)		54	137	63	119	53	29	6
= % (of total nest number)		27%	59%	42%	24%	19%	23%	19%
TU nest (% of total nest number)		8%	5%	3%	2%	2%	7%	23%
Nest abandoned prior nest owner replacement (OA+FOA)		77%	86%	90%	59%	64%	39%	42%
Moving due FU+TU (% of abandoned nests)		6%	6%	5%	7%	2%	7%	17%

Based on the relatively similar character and frequency of different nest owner replacement patterns summarized in Table 2, we assume that the general nest founding strategy of all of the species is in fact very similar, but *Colletes* have remarkably higher incidence of cohabiting of one nest by two females (TU-PE) compared to other species. This result indicates somehow increased tolerance to conspecific females in this species, which we have also personally experienced during field observation. Although we are aware of limited number of observations to support this statement, such higher tolerance might generally represent an example of very important intermediate stage for the evolution of communality and is worth further study (although any sociality is very rare in Colletidae; [35]). Similar accidental joining of a second female to an active nest was also observed in *Sphex ichneumoneus* [18], [19]. Neither the analysis of the other types of behaviour of the original nest owner after owner change nor the behaviour of the original host showed any consistent pattern across different season in the studied species, most likely indicating that there is no common nest owner replacement handling strategy and that the behaviour of both the host and the potential usurper most likely depends on the particular circumstances.

We further quantified the general percentage of nest abandonment followed by founding of a new nest for each species and season. In *Andrena*, nest abandonment occurred in 19–24% of all observed nests; in *Anthophora*, this occurred in 27–59% nests; in *Colletes* this occurred in 23% of nests; and in *Osmia* it occurred in 19% of nests (Table 2). Notably, the reason for nest abandonment and subsequent movement appears to be unknown in most cases (the nests that are gradually abandoned during the season, because their owner died, are not included in this analysis). The usurpations, either successful (TU) or unsuccessful (FU), are responsible for, at most, 33% of the detected cases of nest abandonment in *Osmia* and much less in all of the other species (Table 2). The repeated nest abandonment and founding of new nests during the season without any evident reason is reported in many unrelated bee and wasp species [28], [29], [31], [36]–[40] and seems to be a common feature of solitary bees and wasps. This behaviour might result from an adoption of risk-spreading strategy (bet-hedging) in the places with high parasitic pressure or unstable environmental conditions [39], [41], [42].

Brockman et al. and Brockman and Dawkins [18], [19] showed that *Sphex ichneumoneus* individually chooses between a strategy of “digging” (constructing a completely new nest) and “entering” (inhabiting any existing nest) during each nest founding. They also noted that the necessary condition for this strategy is the constant supply of empty nests during the entire nesting season caused by the frequent abandonment of the active nest. A similar pattern was also observed in *Crabro monticola* [38], and it corresponds well with the observed behavioural patterns of the bees in our study. Thus, we expect that the original facultative nest founding strategy of all of the observed solitary bee species is using of any pre-existing nest, similar to the “entering” strategy of wasps, rather than cleptoparasitism (here usurpations) per se. From this point of view, the observed true usurpations and occasional coexistence of two females within one nest (TU-PE), described above, represent only by-products of this strategy in which a female does not distinguish between an empty nest and an active nest [18], [19].

**Figure 2:**
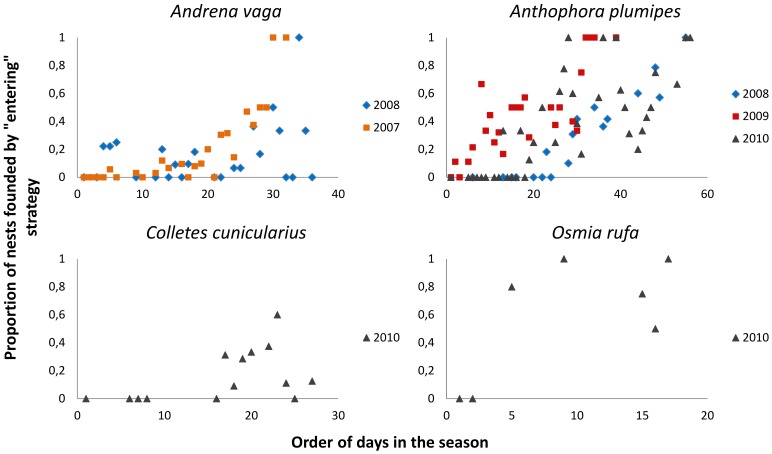
Proportion of nests founded by “entering” strategy each day during the season. The figure shows the results for different species and years.

We examined the distribution of the “entering” strategy during the nesting season of bees because one would expect it to increase with the upcoming end of the season, when the time becomes the limiting factor [43]. We found out that the tendency to found a new nest by utilising any active or abandoned nest increases with the upcoming end of the season in all the species and the relationship is significant in most of them (Fig. 2; Table 3).

**Table 3:** Results of quasibimominal glm model describing dependency of proportion of founding new nest by “entering” strategy on the order of days within the season and results of correlation of the order of days within the season with the number of available empty nests.

		Order of day		Available nests	
Species	*N*	*F*	*P*	*r^2^*	*P*
*Andrena* 2007	26	96.88	<0.001	0.959	<0.001
*Andrena* 2008	26	1.88	0.1985	NA	NA
*Anthophora* 2008	18	58.376	<0.001	0.934	<0.001
*Anthophora* 2009	26	25.845	<0.001	0.985	<0.001
*Anthophora* 2010	38	15.088	<0.001	0.909	<0.001
*Colletes* 2010	14	3.1522	0.1012	NA	NA
*Osmia* 2010	7	4.0307	0.1009	NA	NA

We further detected that the number of abandoned empty nests continually increases during the season and the correlation is strong in the seasons and species where the number of abandoned nests was known (correlation coefficients: *P*<0.001, *r^2^*>0.9, Table 3). We thus assume that the increasing number of available empty nest during the season might be a key factor that enables the increase in adoption of “entering” strategy. Unfortunately, both factors (order of days and number of empty nests) are so closely correlated, that the glm analyses have not enabled us to distinguish which of these factors is proximate.

Our data suggest that the adoption of “digging” or “entering” strategy is not constant but condition-dependent. As mentioned in the introduction, similar condition-dependent strategy was also reported by other researchers in different species [12], [13], [15]–[17]. Notably, and contrary to our results, Brockmann and Dawkins [18] did not find a time-dependent frequency of nests founded by “entering” in *Sphex ichneumoneus*, but they tried to detect it using a different methodology. However, the tendency to enter conspecific nests by old “senile” females at the end of the season was described by Malyshev [44] and Fabre [24] found that the probability of nest usurpation does not decrease with the increasing number of empty nests during the season in *Osmia tricornis*, which is consistent with our results.

**Figure 3:**
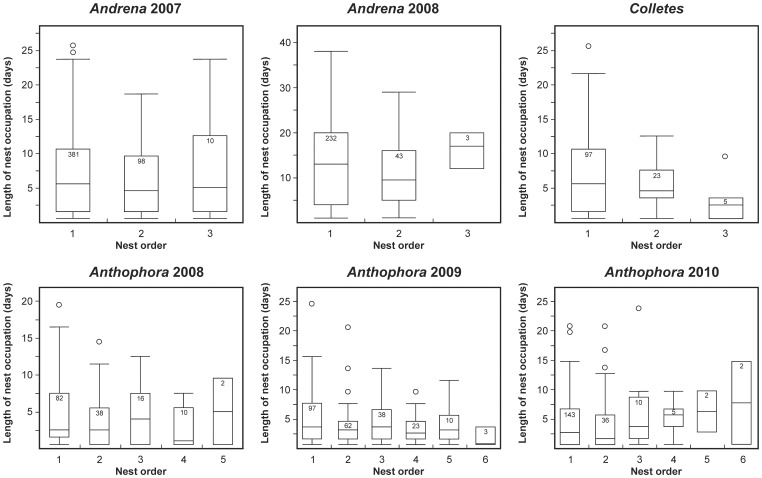
Length of occupation of subsequent nests. The figure shows the results for different species and years. The numbers in the upper part of box-plots indicate the number of cases.

**Figure 4:**
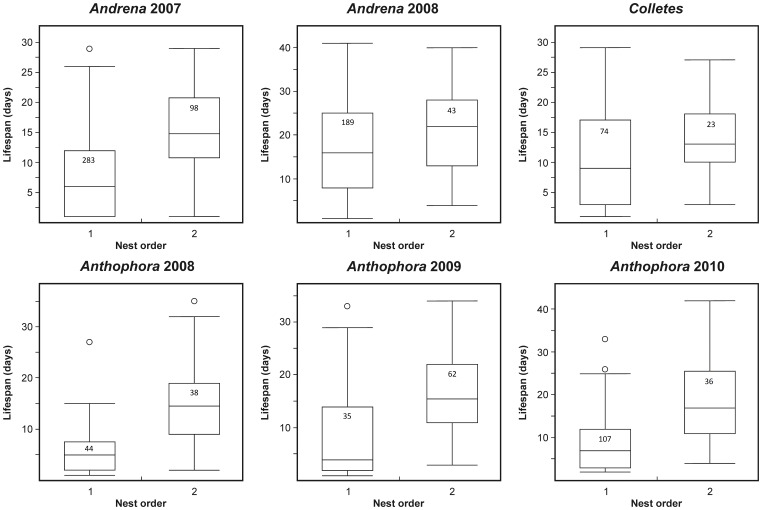
Longevity of females owning one or multiple nests. The figure shows the results for different species and years. 1 = one nest, 2 = multiple nests.

Our results showed that regular nest abandonment and nest owner replacement appears to be common in solitary bees; however, this says nothing regarding why the females should leave their nests. We examined the time spent in each nest and the longevity of females and found that 1) the time spent in the nest, regardless of their order, does not significantly differ between the subsequent nests (one-way ANOVA, *P*>0.05 in all of the studied species except for *Anthophora* in 2009, Table 4, Fig. 3), and 2) the females with more than one nest live significantly longer than females with only one nest (two-tailed standard two-sample t-test, *P*<0.01 in all of the studied species except for *Colletes*, Table 4, Fig. 4). These two results indicate that when females live long enough, they tend to abandon their nests after some critical time and found a new one. This result might be explained by the concept of a risk-spreading strategy, which states, that genotypes with lower variance in fitness should be favoured at the cost of lower mean fitness in a strongly stochastic environment by means of physiology or behaviour that spreads risk of encountering an unfavourable environment over time or space (see Hopper [42] for examples and details). Neff et al. and Williams et al. [29], [45] indeed reported the tendency to construct one-cell nests during heavy rains or attacks from massive amounts of ants in some bee species. The observed pattern of nest abandoning behaviour indicates the likely presence of such risk-spreading strategy in all four studied species, each of which hosts parasites associated to the nesting sites (*Bombylius* sp. in *Andrena*, *Colletes* and *Osmia*, *Nomada lathburiana* in *Andrena*, *Melecta albifrons* in *Anthophora*, *Sphecodes albilabris* in *Colletes* and *Stylops ater* in *Andrena*). Although we do not have the precise data on the rate of parasitism in all the species, we know that these parasites can be variably successful in host infestation in different years depending on the particular climatic conditions (unpublished results). Bulmer [46] used mathematical modelling to show that the costs of nest abandonment outweigh the benefits of risk-spreading when considering that the nest must be constructed and defended, but this study did not include nest usurpation, which helps saving some energy and time.

**Table 4:** Test of the differences in the length of time spent in subsequent nests (One-way ANOVA) and test of the hypothesis that females with more than one nest live significantly longer than females with only one nest (Two-tailed standard two-sample t-test).

	Time in subsequent nests		Longevity and nestcount	
Species	*F*	*P*	*t*	*P*
*Andrena* 2007	0.2214	0.6382	−8.4043	0
*Andrena* 2008	0.2325	0.6300	−3.1206	0.0025
*Anthophora* 2008	0.5389	0.4640	−5.9029	0
*Anthophora* 2009	5.61	0.0204	−5.0357	0
*Anthophora* 2010	2.0849	0.1504	−5.8506	0
*Colletes* 2010	2.90	0.1001	−1.7866	0.0805
*Osmia* 2010	NA		NA	

NA – not analysed. For the number of cases in each species and season see Fig. 3 and Fig. 4.

We assume that the increased turnover of nests at the nesting site due to the adoption of the “entering” strategy must have resulted in increase of intraspecific contacts between females. As mentioned, constructing a nest is always a significant investment and therefore should be protected. Contrary to this expectation, the owners obviously do not invest much energy into nest protection, which results in a low level of conflicts between females. We observed only 13 cases of aggressive behaviour, such as pressing bites and attempts to sting, in all of the species during all of the years of observations, compared with 91 cases of true or failed usurpations, which we take as the minimal estimate of cases, where the intraspecific contact between females must have occurred (Table 5). Because we probably strongly underestimated the total number of possible encounters of two females at the nest entrance by reducing it to the number of TU and FU situations, the true ratio of aggressive and nonaggressive encounters is even much lower. Without the individual marking of females, one could say that no interaction among females or interchange of nests occurred at the observed nesting site. We assume that this is the reason why the nest owner replacements and usurpations are rather neglected in solitary bees. Similarly, low levels of aggressiveness at the intraspecific level during female contact has also been reported in other solitary bees [17], [31]; and unusual tolerance was also reported in some crabronid wasps, e.g. [16], [38], [39]. Described presence of intraspecific cleptoparasitism (here usurpations) and observations of low aggressiveness in bees support the mathematical prediction that the tolerance of cleptoparasitic cheating ( = low level of intraspecific aggressiveness even though the usurpations are commonly present) may occur when the necessary protection against conspecific cleptoparasites is long-term and costly [47]. Because contact between female bees in aggregations are obviously very common, and the fighting between them could be very exhausting (such as in *Hoplitis* [13]), this model is likely an analogy to the war of attrition [48]. Bees have an efficient defence mechanism but do not use it, which makes the neighbourhood society peaceful, although there is a relatively high frequency of cleptoparasitism (nest usurpations).

**Table 5:** Comparison of number of failed and true usurpations (FU+TU) with the number of observed aggressive incidents in all species across all the years of observation.

Species	FU+TU	Aggressiveness
*Anthophora* 2008–2010	38	3
*Andrena* 2007–2008	35	3
*Colletes* 2010	11	4
*Osmia* 2010	7	3
**Total**	**91**	**13**

Our study shows a general picture about the nesting behaviour of solitary bees as the possible analogy to the prior behavioural state for the evolution of alternative life-strategies in bees, such as cuckoo behaviour (obligate brood parasitism), communality and indirectly also eusociality. Positive fitness consequences associated with avoiding excavation costs and diluting parasite pressure due to risk-spreading strategy of solitary bees could represent a parallel to the forces driving the evolution of preadaptations important in the evolution of all recent social and cleptoparasitic bee species in the past. Although the described nesting dynamics with common utilising of pre-existing nests seems to be just an opportunistic behaviour, it makes the contacts between conspecific solitary females very frequent. The relatively high number of detected true usurpations in studied species shows that such contacts are not always amicable. Frequent nest inspections and nest switches may further enhance other cheats like intraspecific cuckoo behaviour, but this behaviour needs to be investigated in more complex studies in the future. On the other hand, usurper accepted by nest owner is practically identical to accepted “joiner” in communal nesting bee [7]. Tolerance of usurpers can thus theoretically represent one of the possible ways towards communality and other types of social behaviour. In fact, our observations of short term coexistence of two females in *C. cunicularius* almost meet a definition of communal nesting [8], which is worth further research.
